# Impact of early mobilization on the duration of delirium in elderly hospitalized patients: A retrospective cohort pilot study

**DOI:** 10.1097/MD.0000000000031641

**Published:** 2022-11-04

**Authors:** Satoshi Anada, Miho Iigaya, Megumi Takahashi, Kazue Soda, Namiko Wada

**Affiliations:** a Department of Rehabilitation, Kitasato University Kitasato Institute Hospital, Tokyo, Japan; b Department of Neurology, Kitasato University Kitasato Institute Hospital, Tokyo, Japan; c Department of Psychiatry, Kitasato University Kitasato Institute Hospital, Tokyo, Japan; d Department of Pharmacy, Kitasato University Kitasato Institute Hospital, Tokyo, Japan; e Department of Nursing, Kitasato University Kitasato Institute Hospital, Tokyo, Japan.

**Keywords:** delirium, early mobilization, rehabilitation, sitting, wheelchair, walking

## Abstract

Development of delirium during hospitalization impairs the activities of daily living in elderly hospitalized patients. In clinical practice, early mobilization from bed is recommended to reduce delirium incidence and hospitalization stay. However, the effects of early mobilization on elderly inpatients with delirium have not been established yet. The aim of this study was to investigate the association between early mobilization and the duration of delirium in elderly inpatients with delirium. This retrospective cohort pilot study examined 45 participants (23 males, 22 females; mean age: 84.5 ± 6.6 years), who developed delirium during hospitalization. Of the participants, 28 were surgically treated and 17 were non-surgically treated. We classified early or delayed mobilization based on the median number of days until the start of mobilization and compared after propensity score matching to adjust for baseline characteristics. Additionally, we examined the correlation between the number of days until the start of mobilization and the duration of delirium. The duration of delirium was significantly shorter in the early mobilization group, particularly in terms of sitting on the bed and wheelchair use than that in the delayed mobilization group {median: 4.0 [interquartile range (IQR): 2.0–6.0] vs 8.0 [IQR: 7.0–14.5] days, *P* = .013; median: 3.0 [IQR: 2.0–5.5] vs 11.0 [IQR: 7.5–14.5] days, *P* = .004, respectively}. Moreover, the duration of delirium significantly positively moderate correlated with the time until the start of sitting on the bed and wheelchair use (Spearman *r* = 0.527; *P* = .012, Spearman *r* = 0.630; *P* = .002, respectively). The results of this study suggest that early mobilization from sitting on the bed or wheelchair use after hospitalization or surgery may shorten the duration of delirium. Because the sample size of this pilot study is small, careful interpretation is needed, and further research is warranted.

## 1. Introduction

Delirium, which commonly occurs in elderly people, is an acute decline in cognitive functioning affecting attention and cognition.^[1]^ Furthermore, delirium during hospitalization has been associated with increased mortality, prolonged hospitalization, and increased institutionalization.^[2]^ In the United States, the 1-year healthcare cost of delirium is estimated to range from $38 billion to $152 billion.^[3]^ The prevalence of delirium in elderly hospitalized patients is 18% and is attributed to an interaction between baseline predisposing factors and precipitating factors that occur during hospitalization.^[4]^ Furthermore, a prospective study found that cognitive decline accelerated and eventually led to dementia even after the resolution of delirium in elderly hospitalized patients.^[5]^ In Japan, the social cost of dementia is estimated at 14.5 trillion JPY, of which 1.9 trillion JPY and 6.4 trillion JPY are for medical care and long-term care, respectively, so it is important to interventions to mitigate this impact should be considered.^[6]^ Another prospective study reported that the development of delirium during hospitalization causes activity of daily living (ADL) impairments in 50.9% and instrumental ADL impairments in 82.5% elderly hospitalized patients.^[7]^ On the contrary, Kiely et al^[8]^ reported that when the duration of delirium was < 2 weeks, ADL recovery was better. In clinical practice, early mobilization from bed is recommended to reduce the incidence of delirium and the length of hospital stay.^[9,10]^ However, evidence demonstrating the early mobilization effects on the duration of delirium among patients is lacking.

Hence, this study proposed to clarify the association between early mobilization and the duration of delirium in elderly hospitalized patients with delirium.

## 2. Methods

### 2.1. Study design and participants

This retrospective cohort pilot study enrolled community-dwelling older adults aged ≥75 years, who were admitted to Kitasato University Kitasato Institute Hospital (Tokyo, Japan) between August 2017 and January 2020, and diagnosed with delirium during their hospitalization for other diseases and injuries.

The inclusion criteria in this study were as follows: community-dwelling older adults aged ≥75 years, admitted to our hospital directly from their homes, and diagnosis of delirium based on the fifth edition of the American Psychiatric Association’s Diagnostic and Statistical Manual of Mental Disorders (DSM-5)^[11]^ criteria during hospitalization. The exclusion criteria were as follows: hospitalization for <2 days, terminal disease (metastatic cancer on palliative care, such as using opioids for pain control), psychiatric disease (undergoing treatment for mental illness with strong positive symptoms prior to hospitalization), severe dementia, and alcohol-related delirium.

This study was conducted in accordance with the guidelines proposed by the Declaration of Helsinki. The Committee of Ethics of Kitasato University Kitasato Institute Hospital reviewed and approved the study protocol (no. 17031), and the study was conducted after obtaining written consent from the patients’ representatives after presenting a full explanation of the study.

### 2.2. Study procedures

#### 2.2.1. Baseline data collection.

We collected baseline data from the patients’ medical records, including age, gender, diagnosis, history of dementia, presence or absence of surgery, Charlson comorbidity index, and medications (use of psychotropics, analgesics, sleep medications, beta-blocker, H2-blocker, steroids, and opioids). The diagnosis was defined as what led to the current hospitalization, history of dementia was defined as what was diagnosed prior to hospitalization, and surgery was defined as what was performed during the current hospitalization.

#### 2.2.2. Mobilization.

Information on time of mobilization following onset of delirium was retrieved from detailed medical records. The number of days until the start of mobilization was counted using the day of delirium as Day 0 based on DSM-5 diagnostic criteria. Moreover, mobilization was classified in the following order: sitting on the bed, wheelchair use, and walking. This study included both cases wherein the patient performed mobilization under staff supervision and cases where the patient was assisted by a physical therapist, occupational therapist, or nurse.

#### 2.2.3. Outcome measures.

In this study, the primary outcome measure was “duration of delirium,” which was assessed by dividing participants into the early and delayed mobilization groups. Delirium was diagnosed by both an experienced neurologist and a psychiatrist diagnosed based on the DSM-5 diagnostic criteria. The duration of the delirium was determined by the complete agreement between the neurologist and psychiatrist’s diagnosis.

Additionally, we investigated the relationship between the time interval until the start of mobilization and the duration of delirium as the secondary outcome measure. Mobilization status was classified as early or late based on the time interval until start of mobilization, with reference to a previous study.^[12]^ A time interval from 0 to the 50th percentile was defined as early mobilization, while a time interval greater than 50th percentile was defined as delayed mobilization.

### 2.3. Statistical methods

#### 2.3.1. Sample size calculation.

In view of findings from previous study in delirious people,^[13]^ we based sample size calculations on the expected duration of delirium. At a significance level of 5% and a power of 80%, with a standard deviation (SD) of 2.3, the sample size required to detect a 2-day difference in change in delirium duration between groups was estimated to be 44 subjects. R version 4.2.1 (R Foundation for Statistical Computing, Vienna, Austria) was used to calculate the sample size.

#### 2.3.2. Propensity score matching.

Propensity score matching methods (one-to-one matching)^[14]^ were performed to adjust for all baseline characteristics. We used multivariate logistic regression to generate the propensity scores, with “early or delayed mobilization” as the outcome and baseline characteristics—age, gender, diagnosis, history of dementia, presence or absence of surgery, Charlson comorbidity index, and medications—as the independent variables. The early and delayed mobilization groups were well matched on the basis of all baseline characteristics. Mobilization was staged into the following 3 categories: sitting in bed, wheelchair use, and walking, with matching at each stage. Early and delayed mobilization participants with similar propensity scores were matched as follows: 11 pairs in “sitting in bed,” 11 pairs in “wheelchair use,” and 9 pairs in “walking.”

#### 2.3.3. Statistical analysis.

All categorical variables are expressed as frequency and percentage, whereas continuous variables are presented as mean and SD or median and interquartile range (IQR), based on the normal distribution assumptions evaluated using the Shapiro–Wilk test.

For the univariate analysis, we used the Student *t* test to compare the mean values and the Mann–Whitney *U* test to compare the median values. Moreover, we compared frequencies and ratios (%) using the chi-squared test. Effect size statistics were calculated using Cohen’s *d* for the Student *t* test, *r* for the Mann–Whitney *U* test and Cramer’s *V* for the chi-squared test. Furthermore, we investigated the relationship between the time interval until the start of mobilization and the duration of delirium using the Spearman correlation coefficient. We considered 2-sided *P* < .05 as statistically significant. The IBM SPSS Statistics version 24.0 (IBM Corp., Armonk, NY) was used to perform all statistical analyses.

## 3. Results

### 3.1. Study population

**Figure 1.**
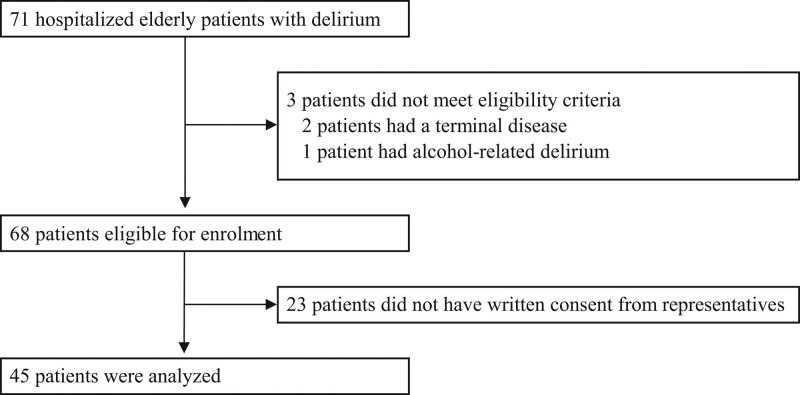
Trial flowchart.

Of the 71 participants who developed delirium during the study period, 3 were excluded (2 patients with terminal disease and 1 with alcohol-related delirium). However, out of the 68 participants who fulfilled the eligibility criteria, 23 did not have written consent from their representatives and were excluded from the analysis (Fig. 1). Thus, we enrolled a total of 45 participants (23 males and 22 females; mean age: 84.5 ± 6.6 years) in this study. The mean duration of delirium was 7.0 days (IQR: 4.0–14.0). The mean times until the start of mobilization were as follows: sitting on the bed, Day 1.0 (IQR: 0.0–2.0); wheelchair use, Day 1.0 (IQR: 0.0–3.0); and walking, Day 4.0 (IQR: 1.0–5.0; Table 1). Therefore, early mobilization was operationally defined as sitting on the bed within 1 day, using a wheelchair within 1 day and walking within 4 days, in the entire study population. The surgically treated patients consisted of 28 participants (10 males and 18 females; mean age: 82.5 ± 5.7 years). Of these, 21 (75.0%) had an orthopedic disease, 5 (17.9%) had cancer, and 2 (7.1%) had a cardiovascular disease. Additionally, 2 (7.1%) participants had a history of dementia. The mean times until the start of mobilization were as follows: sitting on the bed, Day 1.0 (IQR: 0.0–2.0); using a wheelchair, Day 1.0 (IQR: 0.0–2.0); and walking, Day 4.0 (IQR: 1.0–6.0). The non-surgically treated patients consisted of 17 participants (13 males and 4 females; mean age: 87.8 ± 6.9 years). Of these, 2 (11.8%) had orthopedic disease, 4 (23.5%) had cancer, 3 (17.6%) had cardiovascular disease, 3 (17.6%) had respiratory disease and 5 (29.4%) had some other disease. In addition, 4 (23.5%) participants had a history of dementia. The mean times until the start of mobilization were as follows: sitting on the bed, Day 0.0 (IQR: 0.0–1.0); using a wheelchair, Day 2.0 (IQR: 1.0–5.0); and walking, Day 3.0 (IQR: 1.0–5.0).

**Table 1:** Baseline characteristics of participants.

	Total	Non-surgical	Surgical
	*n* = 45	*n* = 17	*n* = 28
**Age, yr, mean ± SD**	84.5 ± 6.6	87.8 ± 6.9	82.5 ± 5.7
**Female, n (%**)	22 (48.9)	4 (23.5)	18 (64.3)
**Diagnoses, n (%**)			
** Orthopedic disease**	23 (51.1)	2 (11.8)	21 (75.0)
** Cancer**	9 (20.0)	4 (23.5)	5 (17.9)
** Cardiovascular disease**	5 (11.1)	3 (17.6)	2 (7.1)
** Respiratory disease**	3 (6.7)	3 (17.6)	0 (0.0)
** Other**	5 (11.1)	5 (29.4)	0 (0.0)
**Dementia, n (%**)	6 (13.3)	4 (23.5)	2 (7.1)
**Charlson comorbidity index >1, n (%**)	18 (40.0)	10 (58.8)	8 (28.6)
**Medications, n (%**)			
** Psychotropics**	28 (62.2)	12 (70.6)	16 (57.1)
** Analgesics**	27 (60.0)	6 (35.3)	21 (75.0)
** Sleep medications**	15 (33.3)	6 (35.3)	9 (32.1)
** Beta-blocker**	7 (15.6)	3 (17.6)	4 (14.3)
** H2-blocker**	5 (11.1)	3 (17.6)	2 (7.1)
** Steroids**	5 (11.1)	2 (11.8)	3 (10.7)
** Opioids**	1 (2.2)	1 (5.9)	0 (0.0)
**Duration of delirium, d, median (IQR**)	7.0 (4.0–14.0)	7.0 (5.0–14.0)	6.5 (3.0–14.0)
**Start of mobilization, d, median (IQR**)			
** Sitting on the bed**	1.0 (0.0–2.0)	0.0 (0.0–1.0)	1.0 (0.0–2.0)
** Wheelchair use**	1.0 (0.0–3.0)	2.0 (1.0–5.0)	1.0 (0.0–2.0)
** Walking**	4.0 (1.0–5.0)	3.0 (1.0–5.0)	4.0 (1.0–6.0)

Non-surgical, participants who did not undergo surgery; Surgical, participants who underwent surgery.

IQR = interquartile range, SD = standard deviation.

### 3.2. Duration of delirium in early versus delayed mobilization

#### 3.2.1. Sitting on the bed.

Table 2 shows the data on the duration of delirium in the early and delayed groups under time until the start of sitting on the bed. Early mobilization was operationally defined as sitting on the bed within 1 day. The baseline characteristics did not significantly differ between the early and delayed mobilization groups after propensity score matching. The duration of delirium was significantly shorter in the early mobilization group than in the delayed mobilization group (4.0 days [IQR: 2.0–6.0] vs 8.0 days [IQR: 7.0–14.5], respectively; *P *= .013; effect size = 0.527).

**Table 2:** Comparing the duration of delirium between early and delayed start of sitting on the bed.

	Unadjusted			After propensity score matching			
	Early mobilization	Delayed mobilization		Early mobilization	Delayed mobilization		
	**(<D 2**)	**(≥D 2**)		**(<D 2**)	**(≥D 2**)		
**Early mobilization vs delayed mobilization**	*n* = 32	*n* = 13	***P* value**	*n* = 11	*n* = 11	***P* value**	**Effect size**
**Age, yr, mean ± SD**	84.2 ± 6.8	85.3 ± 6.2	.612	85.2 ± 7.5	84.4 ± 5.6	.751	0.121
**Female, n (%**)	16 (50.0)	6 (46.2)	.815	6 (54.5)	6 (54.5)	1.000	0.000
**Diagnoses, n (%**)			.728			.630	0.281
** Orthopedic disease**	16 (50.0)	7 (53.8)		8 (72.7)	7 (63.6)		
** Cancer**	7 (21.9)	2 (15.4)		1 (9.1)	2 (18.2)		
** Cardiovascular disease**	3 (9.4)	2 (15.4)		1 (9.1)	0 (0.0)		
** Respiratory disease**	3 (9.4)	0 (0.0)		0 (0.0)	0 (0.0)		
** Other**	3 (9.4)	2 (15.4)		1 (9.1)	2 (18.2)		
**Dementia, n (%**)	5 (15.6)	1 (7.7)	.478	1 (9.1)	1 (9.1)	1.000	0.000
**Surgery, n (%**)	19 (59.4)	9 (69.2)	.537	8 (72.7)	8 (72.7)	1.000	0.000
**Charlson comorbidity index >1, n (%**)	12 (37.5)	6 (46.2)	.591	3 (27.3)	5 (45.5)	.375	0.189
**Medications, n (%**)							
** Psychotropics**	21 (65.6)	7 (53.8)	.460	4 (36.4)	5 (45.5)	.665	0.092
** Analgesics**	19 (59.4)	8 (61.5)	.893	7 (63.6)	8 (72.7)	.647	0.098
** Sleep medications**	13 (40.6)	2 (15.4)	.104	2 (18.2)	2 (18.2)	1.000	0.000
** Beta-blocker**	5 (15.6)	2 (15.4)	.984	1 (9.1)	1 (9.1)	1.000	0.000
** H2-blocker**	3 (9.4)	2 (15.4)	.561	2 (18.2)	2 (18.2)	1.000	0.000
** Steroids**	4 (12.5)	1 (7.7)	.642	0 (0.0)	0 (0.0)	NA	NA
** Opioids**	1 (3.1)	0 (0.0)	.519	0 (0.0)	0 (0.0)	NA	NA
**Duration of delirium, d, median (IQR**)	5.0 (3.0–11.5)	12.0 (7.0–14.0)	.033	4.0 (2.0–6.0)	8.0 (7.0–14.5)	.013	0.527

Early mobilization, that is, when the time interval until the start of mobilization was from 0 to the 50th percentile; Delayed mobilization, that is, when the time interval until the start of mobilization was greater than the 50th percentile.

IQR = interquartile range, NA = not applicable, SD = standard deviation.

#### 3.2.2. Start of wheelchair use.

Table 3 shows data regarding the duration of delirium in the early and delayed groups, based on the time taken until the start of wheelchair use. Early mobilization was operationally defined as wheelchair use within 1 day. The baseline characteristics did not significantly differ between the early and delayed mobilization groups after propensity score matching. The duration of delirium was significantly shorter in the early mobilization group than in the delayed mobilization group (3.0 days [IQR: 2.0–5.5] vs 11.0 days [IQR: 7.5–14.5], respectively; *P *= .004; effect size = 0.611).

**Table 3:** Comparing the duration of delirium between early and delayed start of wheelchair use.

	**Unadjusted**			**After propensity score matching**			
	**Early mobilization**	**Delayed mobilization**		**Early mobilization**	**Delayed mobilization**		
	**(<D 2**)	**(≥D 2**)		**(<D 2**)	**(≥D 2**)		
**Early mobilization vs delayed mobilization**	*n* = 29	*n* = 19	***P* value**	*n* = 11	*n* = 11	***P* value**	**Effect size**
**Age, yr, mean ± SD**	83.2 ± 6.4	86.3 ± 6.6	.129	84.0 ± 5.7	84.1 ± 5.6	.970	0.071
**Female, n (%**)	16 (61.5)	6 (31.6)	.047	3 (27.3)	5 (45.5)	.375	0.189
**Diagnoses, n (%**)			.345			.785	0.220
** Orthopedic disease**	15 (57.7)	8 (42.1)		8 (72.7)	7 (63.6)		
** Cancer**	6 (23.1)	3 (15.8)		2 (18.2)	2 (18.2)		
** Cardiovascular disease**	1 (3.8)	4 (21.1)		0 (0.0)	0 (0.0)		
** Respiratory disease**	1 (3.8)	2 (10.5)		1 (9.1)	1 (9.1)		
** Other**	3 (11.5)	2 (10.5)		0 (0.0)	1 (9.1)		
**Dementia, n (%**)	5 (19.2)	1 (5.3)	.173	1 (9.1)	1 (9.1)	1.000	0.000
**Surgery, n (%**)	18 (69.2)	10 (52.6)	.257	8 (72.7)	8 (72.7)	1.000	0.000
**Charlson comorbidity index >1, n (%**)	9 (34.6)	9 (47.4)	.388	3 (27.3)	4 (36.4)	.647	0.098
**Medications, n (%**)							
** Psychotropics**	18 (69.2)	10 (52.6)	.257	7 (63.6)	6 (54.5)	.665	0.092
** Analgesics**	17 (65.4)	10 (52.6)	.388	7 (63.6)	7 (63.6)	1.000	0.000
** Sleep medications**	10 (38.5)	5 (26.3)	.393	4 (36.4)	4 (36.4)	1.000	0.000
** Beta-blocker**	3 (11.5)	4 (21.1)	.384	0 (0.0)	1 (9.1)	.306	0.218
** H2-blocker**	2 (7.7)	3 (15.8)	.393	2 (18.2)	1 (9.1)	.534	0.132
** Steroids**	2 (7.7)	3 (15.8)	.393	1 (9.1)	1 (9.1)	1.000	0.000
** Opioids**	0 (0.0)	1 (5.3)	.237	0 (0.0)	0 (0.0)	NA	NA
**Duration of delirium, d, median (IQR**)	5.0 (3.0–12.0)	8.0 (6.0–14.0)	.088	3.0 (2.0–5.5)	11.0 (7.5–14.5)	.004	0.611

Early mobilization, that is, when the time interval until the start of mobilization was from 0 to the 50th percentile; Delayed mobilization, that is, when the time interval until the start of mobilization was greater than the 50th percentile.

IQR = interquartile range, NA = not applicable, SD = standard deviation.

#### 3.2.3. Start of walking.

Table 4 presents the data regarding the duration of delirium in the early and delayed groups based on the time until the start of walking. Early mobilization was operationally defined as walking within 4 days. The baseline characteristics did not significantly differ between the early and delayed mobilization groups after propensity score matching. However, the duration of delirium did not differ significantly between the early and delayed mobilization groups.

**Table 4:** Comparing the duration of delirium between early and delayed start of walking.

	**Unadjusted**			**After propensity score matching**			
	**Early mobilization**	**Delayed mobilization**		**Early mobilization**	**Delayed mobilization**		
	**(<D 5**)	**(≥D 5**)		**(<D 5**)	**(≥D 5**)		
**Early mobilization vs delayed mobilization**	*n* = 28	*n* = 17	***P* value**	*n* = 9	*n* = 9	***P* value**	**Effect size**
**Age, yr, mean ± SD**	84.4 ± 7.0	84.8 ± 6.0	.844	81.3 ± 5.2	85.2 ± 4.0	.097	0.841
**Female, n (%**)	14 (50.0)	8 (47.1)	.848	5 (55.6)	4 (44.4)	.637	0.111
**Diagnoses, n (%**)			.304			.793	0.240
** Orthopedic disease**	15 (53.6)	8 (47.1)		3 (33.3)	5 (55.6)		
** Cancer**	7 (25.0)	2 (11.8)		3 (33.3)	2 (22.2)		
** Cardiovascular disease**	1 (3.6)	4 (23.5)		0 (0.0)	0 (0.0)		
** Respiratory disease**	2 (7.1)	1 (5.9)		2 (22.2)	1 (11.1)		
** Other**	3 (10.7)	2 (11.8)		1 (11.1)	1 (11.1)		
**Dementia, n (%**)	5 (17.9)	1 (5.9)	.252	2 (22.2)	1 (11.1)	.527	0.149
**Surgery, n (%**)	18 (64.3)	10 (58.8)	.714	5 (55.6)	5 (55.6)	1.000	0.000
**Charlson comorbidity index >1, n (%**)	12 (42.9)	6 (35.3)	.616	2 (22.2)	3 (33.3)	.599	0.124
**Medications, n (%**)							
** Psychotropics**	20 (71.4)	8 (47.1)	.102	7 (77.8)	6 (66.7)	.599	0.124
** Analgesics**	17 (60.7)	10 (58.8)	.900	4 (44.4)	7 (77.8)	.147	0.342
** Sleep medications**	8 (28.6)	7 (41.2)	.384	3 (33.3)	2 (22.2)	.599	0.124
** Beta-blocker**	4 (14.3)	3 (17.6)	.763	0 (0.0)	1 (11.1)	.303	0.243
** H2-blocker**	3 (10.7)	2 (11.8)	.913	0 (0.0)	1 (11.1)	.303	0.243
** Steroids**	3 (10.7)	2 (11.8)	.913	1 (11.1)	1 (11.1)	1.000	0.000
** Opioids**	0 (0.0)	1 (5.9)	.194	0 (0.0)	0 (0.0)	NA	NA
**Duration of delirium, d, median (IQR**)	5.5 (2.5–12.0)	8.0 (6.0–15.0)	.076	5.0 (2.0–6.0)	8.0 (6.0–15.0)	.062	0.440

Early mobilization, that is, when the time interval until the start of mobilization was from 0 to the 50th percentile; Delayed mobilization, that is, when the time interval until the start of mobilization was greater than the 50th percentile.

IQR = interquartile range, NA = not applicable, SD = standard deviation.

#### 3.2.4. Correlation between time to mobilization and duration of delirium.

Table 5 presents the analyses of the correlation between the duration of delirium and the time until the start of mobilization. The duration of delirium was significantly positively moderate correlated with the time until the start of sitting on the bed (Spearman *r* = 0.527; *P *= .012) and wheelchair use (Spearman *r* = 0.630; *P *= .002).

**Table 5:** Correlation between time taken until the start of mobilization and the duration of delirium.

Start of mobilization	Spearman *r*	*P* value
**Sitting on the bed**	0.527	.012
**Wheelchair use**	0.630	.002
**Walking**	0.469	.050

## 4. Discussion

The results of this study provide evidence that early mobilization is associated with shorter duration of delirium in elderly inpatients with delirium. We found that sitting on the bed early (by the next day after onset of delirium) and wheelchair use early (by the next day after onset of delirium) is associated with shorter duration of delirium in this population. Postoperative delirium often occurs on the first postoperative day,^[15]^ delirium has also been associated with falls,^[16]^ and shortening the duration of delirium may be useful in reducing incidence. The early wheelchair use allows patients to spend time away from the bed, which not only encourages them to eat sitting down and use the toilet but also provides mental stimulation. Moreover, early mobilization not only prevented complications (deep vein thrombosis, pneumonia, pleural effusion, and atelectasis)^[17,18]^ but also improved functional status and the ability to perform ADLs at hospital discharge.^[19,20]^ A previous study^[21]^ reported the association of physical restraint use with persistent delirium, incident delirium and its numerous adverse effects, as well as increased agitation, immobility, functional decline, incontinence, pressure ulcers, asphyxiation, and cardiac arrest. Early mobilization in delirious patients accelerates re-orientation and environmental awareness as well as helps normalize the homeostasis of neurotransmitters, particularly the acetylcholine, dopamine, and gamma-aminobutyric acid, which is achieved by verticalization.^[22]^ Mobilization also promotes natural sleep due to exertion, which is important factor for countering delirium through cerebral reorganization.^[2]^ We hypothesize that increased time spent out of bed may encourage patients to use the toilet when they feel the urge to urinate or have a bowel movement, which, in turn, may reduce the psychological stress of the patient. It is possible that decreased duration of delirium could result in other benefits to the healthcare system, such as reduced burden on hospital staff and reduction of medical costs. Postoperative delirium has been reported to be preventable with appropriate intervention (mobility enhancement, cognitive orientation, therapeutic activities, pain control, simple communication standards and approaches to prevent the escalation of behaviors, and nutritional enhancement and sleep enhancement with nonpharmacological approach) in 40% of participants.^[23]^ Furthermore, delayed mobilization in postoperative patients has been reported to affect post-discharge readmission rates, mortality, and ADL and instrumental ADL disabilities.^[12]^ Therefore, delayed mobilization may lead to adverse events after hospital discharge. On the other hand, no significant difference was found in the duration of delirium between early walking participants in our study. This would suggest the importance of sitting in bed or wheelchair rather than walking early in hospitalization. In addition, this may be due to the small sample size in this pilot study, further validation is needed in a large-scale study with a larger number of participants.

This study has some limitations worth acknowledging. First, this was a retrospective pilot study with a relatively small sample size. The results should thus be interpreted with caution until further, more comprehensive research can be conducted for verification. Although we were able to control for differences in baseline characteristics through propensity score matching, our univariate analysis does not exclude the possible influence of confounding factors such as cognitive, hearing or vision impairment, dehydration, sleep deprivation,^[24]^ severe illness or infection, hip fracture, inadequately controlled pain, depression, renal insufficiency, anemia, hypoxia or hypercarbia, poor nutrition, electrolyte abnormalities, poor functional status, polypharmacy and use of other medications, presence of urinary catheter, urinary retention or constipation, and aortic procedures.^[25]^ Finally, the duration of delirium may be influenced by other factors, such as the severity of delirium, nonpharmacological treatment (care and involvement) and ADL level before hospitalization, which were not assessed in this study.

## 5. Conclusions

This pilot study highlights that early sitting on the bed and early wheelchair use significantly reduced the duration of delirium in the elderly inpatients with delirium. Furthermore, it was found that the number of days to mobilization was associated with the duration of delirium. Larger randomized controlled trials are needed to further investigate the benefits of early mobilization on duration of delirium in the elderly inpatients.

## Acknowledgments

We thank the Geriatric Medical Support Team members and staff of the Department of Rehabilitation of our hospital.

## Author contributions

**Conceptualization:** Satoshi Anada.

**Data curation:** Satoshi Anada, Miho Iigaya, Megumi Takahashi, Kazue Soda, Namiko Wada.

**Formal analysis:** Satoshi Anada, Miho Iigaya, Megumi Takahashi, Kazue Soda, Namiko Wada.

**Supervision:** Miho Iigaya.

**Writing – original draft:** Satoshi Anada.

**Writing – review & editing:** Satoshi Anada, Miho Iigaya, Megumi Takahashi.
